# Targeted Analysis of circRNA Expression in Patient Samples by Lexo-circSeq

**DOI:** 10.3389/fmolb.2022.875805

**Published:** 2022-06-08

**Authors:** Isabel S. Naarmann-de Vries, Jessica Eschenbach, Sarah Schudy, Benjamin Meder, Christoph Dieterich

**Affiliations:** ^1^ Department of Internal Medicine III, Klaus Tschira Institute for Integrative Computational Cardiology, University Hospital Heidelberg, Heidelberg, Germany; ^2^ German Center for Cardiovascular Research (DZHK), Partner Site Heidelberg/Mannheim, Heidelberg, Germany; ^3^ Department of Internal Medicine III, Institute for Cardiomyopathies, University Hospital Heidelberg, Heidelberg, Germany; ^4^ Stanford Genome Technology Center, Stanford University School of Medicine, Stanford, CA, United States

**Keywords:** circRNA, cardiomyopathy, SLC8A1, RBM33, RNA-seq

## Abstract

Recently, circular RNAs (circRNAs) have been extensively studied in animals and plants. circRNAs are generated by backsplicing from the same linear transcripts that are canonically spliced to produce, for example, mature mRNAs. circRNAs exhibit tissue-specific expression and are potentially involved in many diseases, among them cardiovascular diseases. The comprehensive analysis of circRNA expression patterns across larger patient cohorts requires a streamlined and cost-effective workflow designed to meet small input requirements. In this article, we present Lexo-circSeq, a targeted RNA sequencing approach that can profile up to 110 circRNAs and their corresponding linear transcripts in one experiment. We established Lexo-circSeq employing total human heart RNA and show that our protocol can detect depletion of a specific circRNA in hiPSC-derived cardiomyocytes. Finally, Lexo-circSeq was applied to biopsies from patients diagnosed with dilated cardiomyopathy (DCM) and hypertrophic cardiomyopathy (HCM), respectively. Interestingly, our results indicate that circular-to-linear-ratios for circSLC8A1 and circRBM33 are deregulated in cardiomyopathy.

## 1 Introduction

**FIGURE 1:**
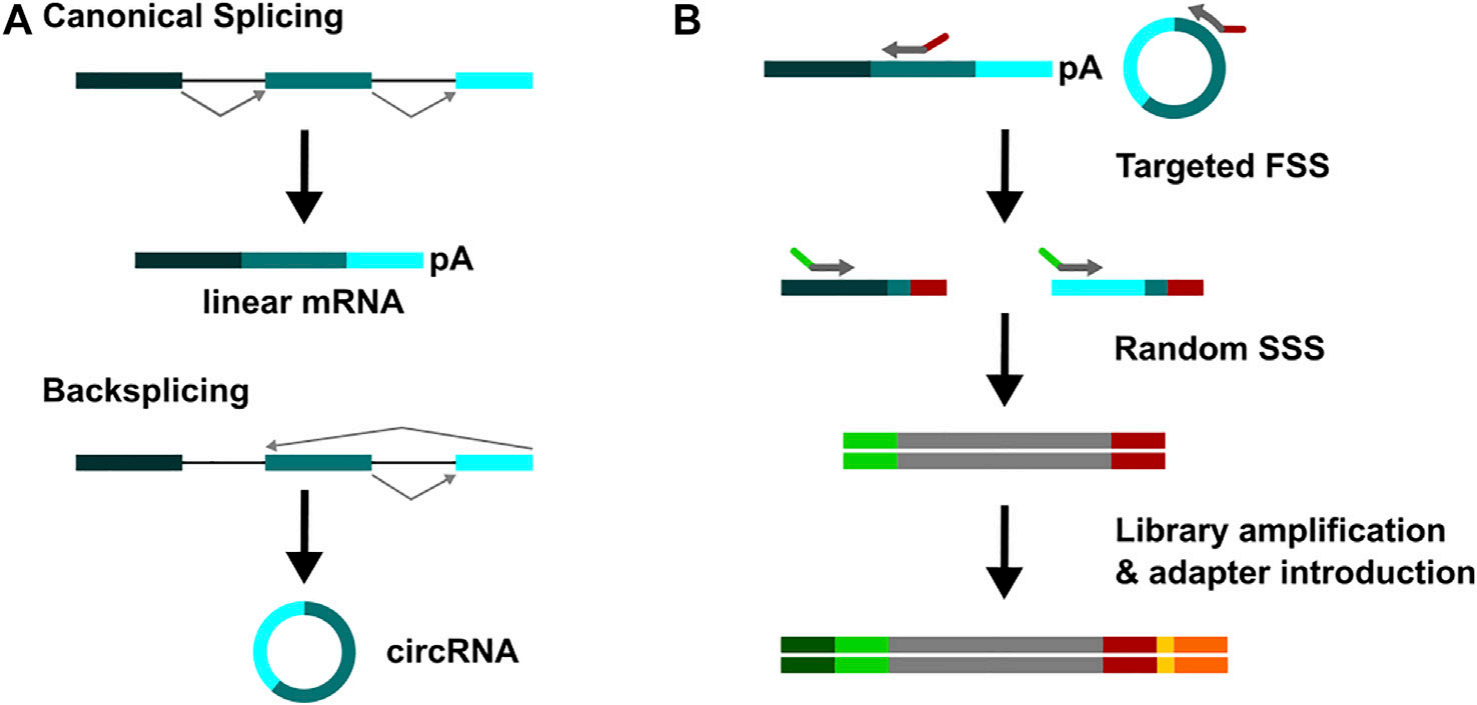
Overview of Lexo-circSeq library preparation. **(A)** Upper panel: The canoncial splicing results in generation of linear mRNAs. Lower panel: circRNAs are generated in a process called back-splicing. **(B)** Total RNA is subjected to targeted First Strand Synthesis (FSS) employing primers directed against up to 110 circRNAs and their corresponding linear transcripts. Random Second Strand synthesis (SSS) enables detection of all 5’ exons. During library preparation, adapters and indices (yellow) for Illumina sequencing are introduced. Here, only one index was used, but dual indexing may be applied as well.

Circular RNAs (circRNAs) are covalently closed RNA molecules. circRNAs in general are generated from protein coding genes by the canonical splicing machinery in a process called backsplicing (Xiao et al., 2020) (Figure 1A). They are typically not polyadenylated due to the lack of free ends and are resistant to exonuclease digestion by RNase R (Suzuki et al., 2006; Salzman et al., 2012). For many circRNAs a cell type and developmental stage specific expression has been described that may be independent of the expression of the corresponding linear host gene (Siede et al., 2017). Functionally, circRNAs seem to be highly diverse. They have, for example, been described as miRNA or protein sponges, regulators of transcription, alternative splicing, and translation (Xiao et al., 2020).

Due to their inherent stability and presence in the blood as well as other body fluids (Bahn et al., 2015; Memczak et al., 2015; Jacky Lam and Dennis Lo, 2019), circRNAs have been proposed as potential biomarkers in a wide range of diseases, such as diverse types of solid cancers and leukemias, neurological disorders, diabetes (Verduci et al., 2021), but also cardiovascular diseases (Lim et al., 2020). The most highly abundant circRNA in cardiomyocytes is circSLC8A1 (Tan et al., 2017), which may sponge miR-133a (Lim et al., 2019) and is thought to be involved in myocardial infarction (Li et al., 2018) as well as myocardial hypertrophy (Lim et al., 2019). Individual studies usually identify a high number of circRNAs, but the overlap between studies is often low. This complicates the identification of relevant circRNAs for functional analyses. We have recently identified a set of 63 positionally conserved circRNAs derived from 50 host genes that are expressed across human, pig, and mouse hearts and enriched by RNase R treatment (Jakobi et al., 2020) that can be used as a resource to study circRNAs in the cardiovascular system. Moreover, whereas many circRNAs are co-expressed with the same profiles as their linear host genes (Tan et al., 2017), we identified hundreds of circRNAs with a host gene-independent expression pattern (Siede et al., 2017). A host gene-independent expression, reflected by changes in the circular-to-linear ratio (CLR), may point to circRNAs with function independent of their host genes.

The high throughput analysis of circRNA expression often requires a large amount of input material and enrichment by RNase R treatment, which displays substantial batch-to-batch variability (Salzman et al., 2012; Zhang et al., 2020; Dodbele et al., 2021). Therefore, after initial identification of a set of highly confident, conserved, and potentially functional circRNAs in a specific organ system or disease context, a targeted approach may be useful for the straight-forward analysis of larger patient cohorts with limited sample availability. Until now, only a protocol has been published for the targeted long read sequencing of a small set of ten circRNAs (circPanel-LRS) (Rahimi et al., 2021).

We present here a method for the targeted detection of a set of up to 110 circRNAs and their corresponding linear transcripts by short read sequencing, which we named Lexo-circSeq. To this end, target specific primers that bind the 3′ exon of the back-splice junction (BSJ) are employed for First Strand Synthesis (FSS) and combined with random primed Second Strand Synthesis (SSS). This enables the detection of all possible 5’ exons, derived from circular as well as linear transcripts. We established Lexo-circSeq for the set of 63 positionally conserved cardiac circRNAs (Jakobi et al., 2020), employing total human heart RNA, hiPSC-CM, and cardiac biopsies from cardiomyopathy patients as model systems. Focusing on the CLR as read out, we identified circSLC8A1 and circRBM33 as candidate deregulated circRNAs in cardiomyopathies.

## 2 Materials and Methods

### 2.1 Culture and Transfection of hiPSC-CM

WT1.14 human induced pluripotent stem cells were differentiated based on described protocols (Lian et al., 2013; Burridge et al., 2014) as described previously (Naarmann-de Vries et al., 2021). On day 23 of the differentiation, 750,000 cells per six well were plated and used on day 29 for transfection of siRNAs with Lipofectamine RNAiMAX (#13778-100, Thermo Fisher Scientific, Waltham, MA, United States). Briefly, 150 µL OptiMEM (Thermo Fisher Scientific) was mixed with 9 µL of Lipofectamine RNAiMAX. In parallel, 150 µL of OptiMEM was mixed with 3 µL of a 10 µM siRNA stock. Both were combined and incubated for 5 min at room temperature before adding 250 µL transfection complex dropwise to hiPSC-CM in fresh cardio culture medium. The transfection complex was removed 24 h post transfection and the cells were harvested 48 h post transfection. SiRNA sequences are listed in Supplementary Table S1.

### 2.2 Patient Samples

Heart biopsies from patients diagnosed with dilated cardiomyopathy (DCM) or hypertrophic cardiomyopathy (HCM) were obtained from the Heidelberg Cardio Biobank (HCB), approved by the ethics committee (Application No. S-390/2011). Samples from heart transplanted (HTX) patients obtained from the HCB were used as controls. The study was conducted according to the principles outlined in the Declaration of Helsinki. All participants have given written informed consent to allow for molecular analysis. Only samples from male patients with an age range of 45–65 years were included. Subject to availability, either one or two biopsies were used for the isolation of total RNA as described below after homogenization.

### 2.3 Isolation of RNA

Total human heart RNA (THHR) (#636532) was purchased from Takara Bio (Kusatsu, Japan). RNA from hiPSC-CM was isolated using the miRNeasy Tissue/Cells Advanced Mini kit (#217604, Qiagen, Hilden, Germany) according to the manufacturers’ protocol. RNA from human heart biopsies was isolated using Trizol. Samples were homogenized in 750 µL Trizol and incubated for 5 min at room temperature before the addition of 200 µL chloroform. Samples were centrifuged (20 min, 10,000 g, room temperature) and the aqueous phase re-extracted with one volume of chloroform: isoamylalkohol (24:1) (5 min, 10,000 g, room temperature). The RNA in the aqueous phase was precipitated with one volume of isopropanol (30 min, 20,800 g, 4°C) in the presence of glycogen (1 µL of 20 mg/ml stock solution, #10901393001, Roche Diagnostics, Penzberg, Germany), washed twice with 1 ml 80% ethanol in DEPC-H_2_O and dissolved in 25 µL DEPC-H_2_O (10 min, 55°C, shaking).

### 2.4 RNase R Digestion

6 µg THHR was digested with 6 U of RNase R for 30 min at 37°C. The enzyme was inactivated by a 10 min incubation at 65°C. A mock reaction (-RNase R) was performed in parallel. After the reaction, 5 ng *in vitro* transcribed *lacI* RNA was added to the reactions as an external control RNA and the reactions purified with Trizol as described above.

### 2.5 Generation of External Control RNA

The generation of *lacI* as an external control RNA has been described previously (Naarmann-de Vries et al., 2021). Briefly, the *lacI* sequence was amplified from pET16b using Q5 polymerase (New England Biolabs, Ipswich, MA, United States) and a forward primer bearing the T7 promoter sequence. *In vitro* transcription reactions were carried out using the T7 Megascript kit (Thermo Fisher Scientific) and purified with RNA Clean and Concentrator kits (Zymo Research, Irvine, CA, United States).

### 2.6 RT-qPCR Analysis

After digestion with RNase R, equal volumes were subjected to reverse transcription using the Maxima H Minus First Strand cDNA Synthesis Kit (#K1682, Thermo Fisher Scientific, Waltham, MA, United States) with Random Primers according to the manufacturers’ protocol. RNA was DNase digested in a total volume of 10 µL for 2 min at 37°C. Reactions were filled up to 15 µL with 1 µL of random primer and 1 µL dNTP solution and denatured (5 min, 65°C). The reaction was completed by addition of 4 µL 5×RT buffer and 1 µL Maxima enzyme and incubated 10 min at room temperature followed by 30 min at 50°C, and denaturation (5 min, 85°C).

For standard qPCR analysis, reverse transcription reactions were diluted 1:20 and used for qPCR analysis on a Viia7 instrument (Thermo Fisher Scientific) with PowerUp SYBR Green PCR Master Mix (Thermo Fisher Scientific) and primers listed in Supplementary Table S2. Whenever possible, primers were designed with a length of 20 nts and a GC content of 50% (melting temperature around 55–57°C, derived from Snapgene Viewer (GSL Biotech LLC, San Diego, CA, United States)). In the case of exons with a low GC content, the primer length was adjusted to meet a melting temperature of 55–57°C. qPCR data were normalized using the ΔΔC_t_ method (Schmittgen and Livak, 2008).

### 2.7 Design of Target-specific First Strand Synthesis Primers

Target-specific reverse transcription (First Strand Synthesis) primers were designed according to the guidelines outlined in the QuantSeq Flex manual. All primers were composed of a partial Illumina P7 adapter extension followed by the target-specific sequence. In the optimal case, primers should be 45 to 50 nts in length. Here, the primer length was in the range of 43–48 nts, which was required to match the optimal melting temperature (Tm). The Tm should be as close as possible to the reaction temperature of 50 °C (±2°C). Tms were calculated with an IDT online tool assuming 75 mM Na^+^, 3 mM Mg^2+^, 0.5 mM dNTPs, and 0.0125 µM oligo. Here, the Tm range was 48.3–52.5°C. If possible, target-specific first Strand synthesis primers were chosen at the binding site of the standard qPCR reverse primer. All target-specific first strand synthesis primers can be found in Supplementary Table S3.

### 2.8 Lexo-circSeq Library Preparation

Libraries were prepared using the QuantSeq-Flex Targeted RNA-Seq Library Prep Kit V2 with First Strand Synthesis Module (Lexogen, Vienna, Austria). Primers were mixed at an equimolar ratio (except for SLC8A1_2/3) to yield a 100 nM primer stock solution. For SLC8A1_2/3, the amount was reduced to 1% of the other primers because of the high abundance of this circRNA. For First Strand Synthesis, 5 µL of primer mix and 250 ng of total RNA were adjusted to 10 µL with H_2_O, denatured for 3 min at 85°C, and afterwards cooled to 50°C. A master mix of 5 µL FS1x, 4.5 µL FS2x, and 0.5 µL E1 enzyme per reaction was incubated for 3 min at 50°C. RNA-primer mix and master mix were mixed by pipetting and incubated for 60 min at 50°C. Template RNA was removed by a 10 min incubation with RNA removal solution at 95°C. The reaction was cooled to 25°C and immediately subjected to random-primed second strand synthesis. 10 µL of SS1 was added. Reactions were incubated 1 min at 98 °C, cooled down to 25 °C in 0.5°C/s increments and incubated 30 min at 25°C. After addition of a mixture of 4 µL SS2 and 1 µL E2 enzyme, the reaction was incubated for an additional 15 min at 25°C. The libraries were purified with the Purification Module with Magnetic Beads (Lexogen, Vienna, Austria). 16 µL magnetic beads (PB) were added to the Second Strand Synthesis reaction and incubated for 5 min at room temperature. After collection of the beads on a magnet, the supernatant was removed and discarded. Beads were resuspended in 40 μL of EB and incubated for 2 min at room temperature. After the addition of 56 μL PS and another incubation (5 min, room temperature), beads were collected on a magnet. The supernatant was removed and discarded. The beads were washed twice with 120 µL 80% ethanol and dried for 5–10 min at room temperature. Beads were resuspended in 21 μL EB and incubated for 2 min at room temperature. After collecting the beads on a magnet, 20 µL were transferred to a new tube and stored at −20°C.

### 2.9 Lexo-circSeq Library Amplification

The optimal cycle number for library amplification was determined by qPCR with the PCR Add-on Kit for Illumina (Lexogen, Vienna, Austria) as described in the manufacturer’s protocol. For the final amplification, 20 cycles were chosen according to the following program: initial denaturation (98°C, 30 s), amplification cycles (98°C, 10 sec; 65°C, 20 sec; 72°C, 30 s), and final elongation (72°C, 1 min). The PCR reaction was set up with 17 µL of library, 5 µL of i7 index primer, 7 µL of PCR mix, and 1 µL of E3 enzyme. Amplified libraries were purified with the Purification Module with Magnetic Beads (Lexogen, Vienna, Austria). 30 µL of magnetic beads (PB) were added to the PCR reaction and incubated for 5 min at room temperature. After collection of the beads on a magnet, the supernatant was removed and discarded. Beads were resuspended in 30 μL EB and incubated for 2 min at room temperature. After the addition of 30 μL PS and another incubation (5 min, room temperature), beads were collected on a magnet. The supernatant was removed and discarded. The beads were washed twice with 120 µL of 80% ethanol and dried for 5–10 min at room temperature. Beads were resuspended in 20 μL of EB and incubated for 2 min at room temperature. After collecting the beads on a magnet, 17 µL were transferred to a new tube and stored at −20°C.

### 2.10 RNA Sequencing and Data Analysis

Lexogen libraries were pooled and sequenced on a MiSeq instrument (Illumina, San Diego, CA, United States) with a MiSeq Reagent Kit v2 or v2 Micro (SR300). All RNA-seq libraries generated for this study are summarized in Supplementary Table S4. Read processing (adapter removal and quality clipping) was performed with Flexbar 3.5 (Roehr et al., 2017), and subsequent read mapping was done with the STAR alignment software (Dobin et al., 2013). Raw read numbers for linear and chimeric reads were used to calculate reads per million uniquely mapped reads (RPM) and the circular-to linear ratio (CLR) as [circ/(circ + lin)], which are listed in Supplementary Table S5-S7. Coverage of individual circRNAs was visualized with the Integrated Genome Viewer (IGV) (Thorvaldsdóttir et al., 2013).

### 2.11 Statistical Analysis

Correlation was analyzed using the Pearson correlation coefficient in Graph Pad Prism (San Diego, CA, United States). Significance levels were defined as * = *p* < 0.05, ** = *p* < 0.01, *** = *p* < 0.001 and are included in the respective figure legends.

## 3 Results

### 3.1 Targeted Detection of circRNAs With Lexo-circSeq

#### 3.1.1 Principle of the Method

Recently, circRNAs have gained a lot of attention, and many studies have identified circRNAs in various settings. However, insight into the function of most circRNAs and their contribution to disease pathogenesis is lagging behind, in particular due to the low abundance of most circRNAs. We present here a method for the targeted sequencing of circRNAs and their corresponding linear transcripts. Lexo-circSeq uses primers that bind on the 3′ site of the (BSJ) (Figure 1B) for First Strand Synthesis (FSS). Reads are thus covering either the BSJ or the canonical linear splice junction (Figure 1A). This targeted FSS is combined with random-primed Second Strand Synthesis (SSS) to allow capture of all possible 5′ exons. During library amplification, i5 indices and adapters are introduced for Illumina sequencing (Figure 1B). With this setup, a panel of up to 110 circRNAs and their corresponding linear counterparts can be analyzed in one sequencing reaction. Linear (aligned) and circular (chimeric) reads are mapped with STAR. Depending on the required read out, either abundance (reads per million, RPM) or circular-to-linear ratio (CLR) may be considered for downstream analysis.

#### 3.1.2 Lexo-circSeq Setup for Conserved Cardiac circRNAs

Recently, we described a set of 63 positional conserved circRNAs in human, pig, and mouse hearts (Jakobi et al., 2020) derived from 50 host genes. Among them are well described circRNAs such as circSLC8A1 (circNCX1) and circHIPK3. All circRNAs were detectable by RT-qPCR in total human heart RNA (THHR) and are resistant to RNase R digestion, whereas most linear RNAs were degraded by RNase R (Supplementary Figure S1). Our aim was to analyze these circRNAs in cardiac and disease-associated samples employing Lexo-circSeq. To this end, target-specific reverse transcription (RT) primers were designed for all 63 circRNAs. As some of the circRNAs are derived from the same host gene and share the same 3′exon, a total of 52 primers were included in FSS (see Supplementary Table S3). To control the efficiency of the target-specific FSS primers, Lexo-circSeq libraries were analyzed by qPCR (Supplementary Figure S2A,B). The C_t_ values of standard qPCR analysis and qPCR analysis employing the target-specific FSS primer showed a significant correlation (Supplementary Figure S2C), indicating that they bind their circRNA targets with a similar efficiency as regular primers lacking the partial Illumina P7 adapter extension.

**FIGURE 2:**
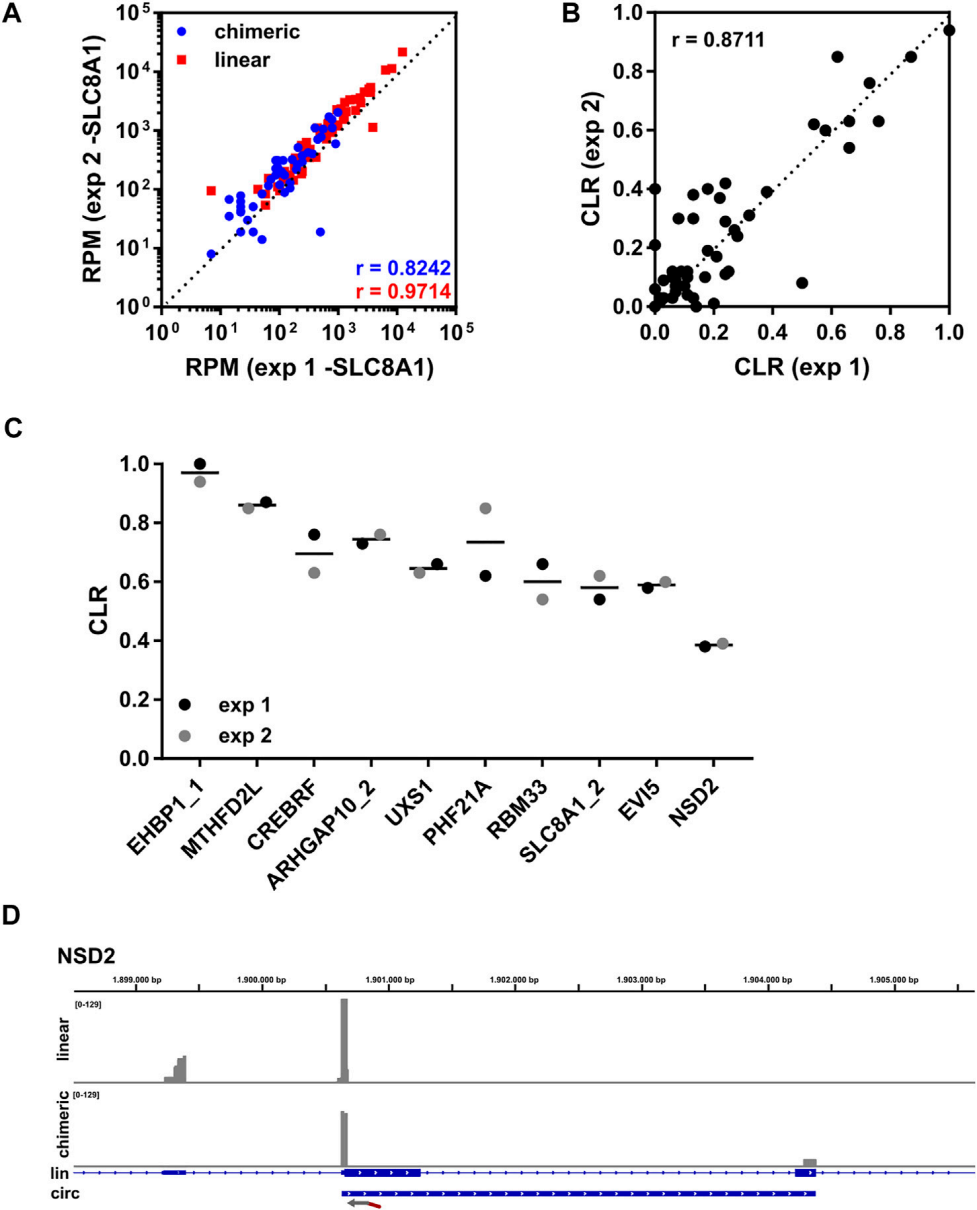
Lexo-circSeq analysis of THHR. **(A)** Correlation of RPM (uniquely mapped reads) for chimeric (blue) and linear (red) reads from experiments 1 and 2 using 250 ng of total RNA as input. circSLC8A1 was excluded from this analysis. r = Pearson correlation coefficient. p < 0.001. **(B)** The CLR was calculated for all targeted circRNAs. Correlation of experiments 1 and 2. r = Pearson correlation coefficient. p < 0.001. **(C)** CLR for the top 10 circRNAs with the highest circular-to-linear ratio. **(D)** Read coverage of the targeted region in NSD2 from exp2. Linear reads are shown in the upper panel, chimeric reads are shown in the lower panel. The exon structure of the NSD2 mRNA and coordinates of the conserved circRNA are depicted below. Both panels were scaled to a data range of 0-129 reads. The binding site of the FSS primer is indicated by an arrow.

Next, we sequenced two independent libraries generated from THHR. Both libraries were prepared from 250 ng total RNA as input (Figure 1), which was determined to be sufficient for detection of all tested circRNAs. Abundant circRNAs were detectable with as little as 10 ng of total RNA. Higher input quantities did not improve results (data not shown). Because we noticed a very high read number for circSLC8A1_2/3 (herein named circSLC8A1) in experiment 1, the respective primer was reduced to 1% in experiment 2. Importantly, the relative proportion of chimeric reads was comparable between the two experiments (Supplementary Figure S3B), although the obtained read number was higher in experiment 2 (Supplementary Figure S3A). The RPMs for linear as well as chimeric reads showed a significant but moderate correlation between experiments (Supplementary Figure S3C). The weakness of correlation was mainly driven by circSLC8A1, as correlation was enhanced significantly after the removal of circSLC8A1 (Figure 2A), indicating an overall good correlation between experiments. We then calculated the CLR for all targeted circRNAs (Figure 2B), and importantly, found a strong correlation between experiments. Figure 2C shows the top 10 circRNAs with the highest CLR. With the exception of circPHF21A, the variation of CLR between experiments is low. All the CLR ratios for this experiment can be found in Supplementary Table S5. Figure 2D shows the exemplary coverage of NSD2. The direction and location of the targeted primer are indicated by an arrow. Whereas the linear reads (upper panel) extend to the 5′exon, chimeric reads (lower panel) extend to a 3′exon that is usually included in the mature circRNA sequence (Figure 2D).

### 3.2 Depletion of SLC8A1 as Proof of Principle

**FIGURE 3:**
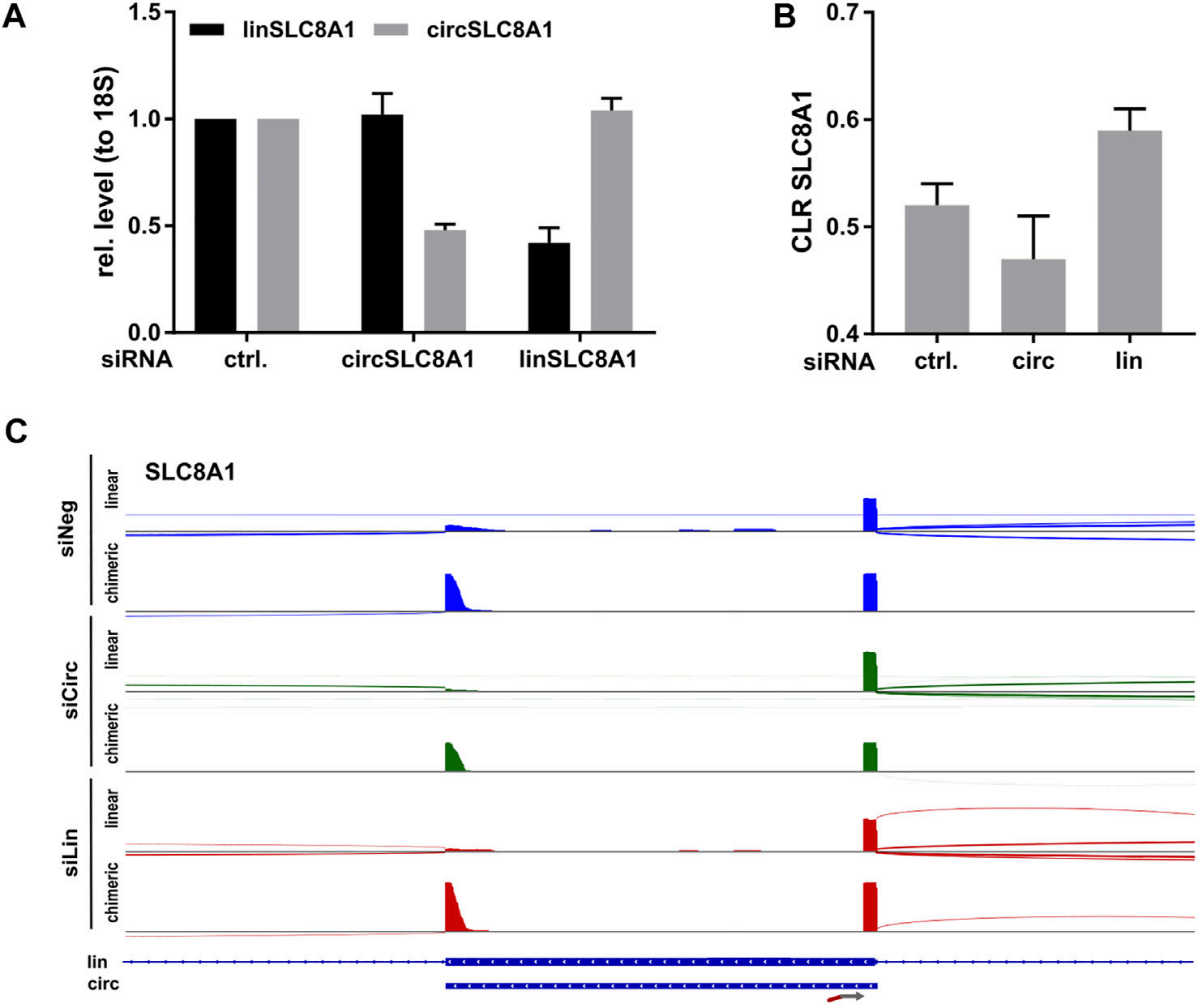
Lexo-circSeq analysis of SLC8A1-depleted hiPSC-CM. hiPSC-CM was transfected with siRNAs targeting circSLC8A1, linSLC8A1 or a non-targeting siRNA (ctrl.) as control. Cells were analyzed 48 h post transfection **(A)** RT-qPCR analysis of linSLC8A1 and circSLC8A1. Data were normalized to 18S and the mean of the control samples. n = 2. **(B)** CLR for SLC8A1 from Lexo-circSeq analysis as indicated. n = 2. **(C)** Sashimi plot of the targeted region in SLC8A1. Linear reads are shown in the respective upper panels, chimeric reads in the lower panel. The different experimental conditions are indicated by color. The exon structure of the SLC8A1 mRNA and coordinates of the conserved circRNA are depicted below. All panels were scaled to a data range of 0-404 reads. The binding site of the FSS primer is indicated by an arrow.

The analysis of THHR showed consistent and reproducible detection of the conserved set of cardiac circRNAs. Next, we performed Lexo-circSeq in human induced pluripotent stem cell-derived cardiomyocytes (hiPSC-CM) as a model system for cardiac development and disease pathogenesis. As a proof of principle, we depleted circSLC8A1 or the corresponding linear transcript (linSLC8A1) from hiPSC-CM employing an siRNA-mediated knock down. RT-qPCR analysis revealed a reduction of both circSLC8A1 and linSLC8A1 below 50% (Figure 3A). Duplicate samples were sequenced according to the established Lexo-circSeq protocol. Raw reads, RPM, and CLR for all targets can be found in Supplementary Table S6. Consistent with the qPCR results, the CLR is reduced in circSLC8A1 depleted cells and increased in linSLC8A1 depleted cells (Figure 3B). Sashimi plots illustrate the linear and chimeric reads for circSLC8A1 (Figure 3C). The linear reads extend to a 3′exon, whereas the chimeric reads show circularization of the depicted exon. The majority of analyzed circRNA/linRNA pairs seems not to be affected by the SLC8A1 depletion (Supplementary Figure S4A,B); however, the CLR for ZEB1 and LRCH1 is interestingly reduced in linSLC8A1 depleted cells (Supplementary Figure S4C,D). Furthermore, circPAN3 seems to be reduced in both circSLC8A1 and linSLC8A1 depleted cells (Supplementary Figure S4E). Further studies are required to determine the significance of these tentative findings and to elucidate whether they indicate a biological function or are an off-target effect of the siRNA-mediated depletion.

### 3.3 Identification and Analysis of Cardiomyopathy Associated circRNAs

The low abundance of circRNAs and the requirement to enrich for circRNAs prior to sequencing often precludes a detailed analysis of limited sample material. Lexo-circSeq can be easily applied to screen a cohort of patient samples for a disease-specific panel of circRNAs with a decent quantity of input material. Here, we exemplify this approach and analyze expression and CLR of the cardiac-conserved circRNA set in a small set of samples from patients suffering from dilated cardiomyopathy (DCM) and hypertrophic cardiomyopathy (HCM) in comparison to heart-transplanted patients (HTX) as a control. The analysis of RNA from patient-derived material is often complicated by low or at least highly variable RNA quality, which may affect the sequencing outcome. In line with this, the percentage of uniquely mapped reads showed variability between samples (Supplementary Figure S5A). However, the fraction of chimeric reads among the uniquely mapped reads was consistent between samples (Supplementary Figure S5B), enabling the analysis of circRNA expression and CLR.

**FIGURE 4:**
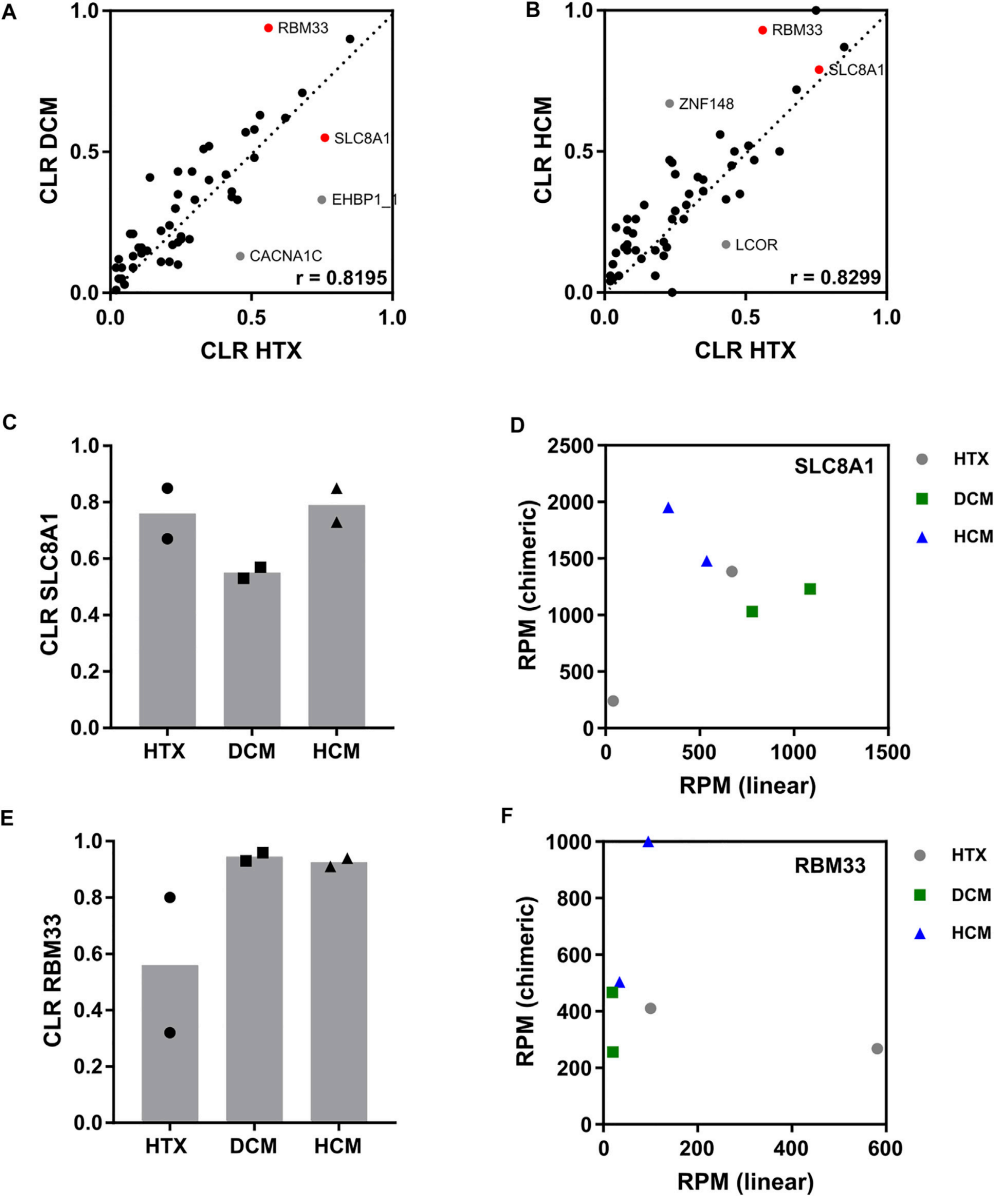
Identification of deregulated circRNAs in cardiomyopathies. Samples from patients diagnosed with DCM, HCM, or HTX as control (two per condition) were analyzed by Lexo-circSeq. **(A)** Correlation of the mean CLR of HTX versus DCM. r = Pearson correlation coefficient. p < 0.001. Candidate deregulated circRNAs are highlighted in red. circRNAs highlighted in grey were excluded as candidates due to low expression. **(B)** Correlation of the mean CLR of HTX versus HCM. r = Pearson’s correlation coefficient. p < 0.001. Candidate deregulated circRNAs are highlighted in red. circRNAs highlighted in grey were excluded as candidates due to low expression. **(C)** CLR for SLC8A1 in HTX, DCM, and HCM samples, as indicated. **(D)** Analysis of SLC8A1 RPM for linear (x-axis) and chimeric (y-axis) reads in HTX, DCM and HCM samples. **(E)** CLR for RBM33 in HTX, DCM and HCM samples as indicated. **(F)** Analysis of RBM33 RPM for linear (x-axis) and chimeric (y-axis) reads in HTX, DCM and HCM samples.

Here, we focused on differences in the CLR. For the two samples per disease condition, correlation was moderate (Supplementary Figure S5C), which was expected from non-related patient samples. For most analyzed circRNAs, the CLR was highly consistent between conditions and no prominent differences were observed (Figure 4A,B). However, we also noticed remarkable exceptions. The level of circSLC8A1 relative to linSLC8A1 is decreased in DCM compared to HTX control (Figure 4C). This seems to be based on reciprocal changes in expression of the linear and circular isoforms (Figure 4D). On the other hand, the CLR of circRBM33 was increased in both DCM and HCM compared to HTX (Figure 4E), which is mainly driven by a lower expression of linear RBM33 transcripts (Figure 4F). Both circRNAs are well supported in terms of raw read numbers. For other circRNAs such as EHBP1_1, CACNA1C, ZNF148, and LCOR, we noticed that the apparently high changes in CLR were due to the low expression of these circRNAs (Figure 4A,B, Supplementary Table S7).

## 4 Discussion

In recent years, circRNAs have become a focus of RNA research. Numerous studies analyzed the expression of circRNAs by high throughput RNA-sequencing (Chen, 2020; Xiao et al., 2020). However, although the function of a few circRNAs was analyzed in detail, knowledge on the function of most circRNAs and a comprehensive view of their contribution to disease pathogenesis is still lacking (Li et al., 2019; Dodbele et al., 2021).

To gain insight into the dynamics of circRNA expression, methods are required that allow the specific and cost-effective analysis of larger sample sets, such as patient-derived cohorts. We present here a targeted RNA-sequencing approach that, based on an initial transcriptome-wide identification of relevant circRNAs, enables the quantification of a panel of up to 110 circRNAs and their corresponding linear transcripts with a low amount of input material. With 250 ng total RNA input material, we were able to detect all targeted circRNAs (Figure 2). We noticed that circRNAs with a relatively high abundance can be detected even with as little as 10 ng of total RNA, which was the lowest quantity tested (data not shown). These input requirements are in a similar range as a published targeted long read sequencing protocol (Rahimi et al., 2021). However, the circPanel-LRS protocol was validated only for the analysis of 10 circRNAs.

In contrast to circPanel-LRS, the Lexo-circSeq approach does not aim at the characterization of full length circRNA sequences but rather at the detection of all 5′ splicing events (back splicing and canonical linear splicing) for a targeted 3′exon (see Figure 1). With this approach, both the expression level of targeted circRNAs as well as the circular-to-linear ratio (CLR) can be determined. Many protocols used to characterize circRNAs rely on RNase R to digest linear transcripts. However, a high variability of RNase R efficiency and specificity has been reported in the literature (Salzman et al., 2012; Zhang et al., 2020; Dodbele et al., 2021) and was also observed by us. This may introduce a significant bias in the quantification of circRNA expression. Our approach does not rely on RNase R digestion, allowing a more accurate quantification of circRNA levels. Both currently available targeted circRNA sequencing protocols, circPanel-LRS (Rahimi et al., 2021) and Lexo-circSeq, introduced here, rely on the preceding identification and characterization of candidate circRNAs by transcriptome-wide untargeted approaches. Also, for the unbiased analysis of larger circRNA sets, a transcriptome-wide approach may be more suitable. With its targeted design, Lexo-circSeq aims to fill a gap between these high-throughput and single target methods.

In a pilot analysis based on two patient samples per disease entity presented here, we provide evidence that the relative proportion of circSLC8A1 is decreased in the analyzed DCM patients, whereas circRBM33 is deregulated in DCM as well as HCM (Figure 4). Given the high natural variability between individual patient samples and the small sample size analyzed here, additional research employing larger patient cohorts is required to provide definitive evidence for these conclusions. circRBM33 was found to be up-regulated in gastric cancer (Wang et al., 2020), cervical cancer (Ding et al., 2021) and abdominal aortic aneurysm (Wang et al., 2021), but a function in cardiomyopathy progression has not been reported until now. In contrast, circSLC8A1 has been previously described to be implicated in CVD (Li et al., 2018; Lim et al., 2019), although its function remains a matter of debate. We observe here a reduced CLR in DCM patients, whereas other studies also found an increase (Siede et al., 2017; Lei et al., 2018) or unchanged CLR (Tan et al., 2017). Further work based on larger patient cohorts is required to elucidate the function of circSLC8A1 in DCM and other CVDs.

We established and validated here the Lexo-circSeq method employing duplicate samples from total human heart RNA, hiPSC-CM, and cardiac biopsies. This analysis revealed a robust and sensitive detection of a set of conserved cardiac circRNAs, but larger cohorts will be required to elucidate circRNA-related regulatory functions. The Lexo-circSeq method can be easily adapted for other biological questions or disease entities. The only prerequisite is a previously defined circRNA set that may have been determined employing non-targeted RNA-sequencing approaches. The input requirements can be scaled according to the expression level of the analyzed circRNAs. The provided primer design criteria should be followed to ensure the efficiency of the target specific primers. Furthermore, we recommend placing the primers at least 20 (better 30–50 nts) apart from the BSJ to ensure proper classification of BSJ reads. In the future, Lexo-circSeq may be used to define specific circRNA signatures that may be used as diagnostic and predictive biomarkers. The transfer to analysis of body fluids such as blood would then enable the simple screening of patient cohorts.

## Data Availability

The datasets generated for this study can be found at BioProject PRJNA825392 (https://www.ncbi.nlm.nih.gov/bioproject/PRJNA825392).
