# Supplementary material for: Targeted Analysis of circRNA Expression in Patient Samples by Lexo-circSeq
Source: Front Mol Biosci. 2022 Jun 8;9:875805. doi: 10.3389/fmolb.2022.875805 (PMC9214859; doi:10.3389/fmolb.2022.875805)
Supplement: Supplementary file 4 [file DataSheet1.PDF]

Supplementary Material

1 SUPPLEMENTARY TABLES AND FIGURES

1.1 Supplementary Tables

Name	sense	antisense
**circSLC8A1**	GAUGAAAUUGUUAGGUUGUtt	ACAACCUAACAAUUUCAUCat
**linSLC8A1**	GCAACGCGGUGAAUGUCUUtt	AAGACAUUCACCGCGUUGCtg

Table S1. siRNA sequences used in this study. As negative control a pre-designed control siRNA was used (Silencer™ Select Negative Control No. 1 siRNA (#4390843, Thermo Fisher Scientific).

These Tables are supplied as individual files:

Table S2. qPCR primers used in this study.

Table S3. Target specific FSS primers.

Table S4. Overview of sequencing runs generated for this study.

Table S5. Sequencing results THHR.

Table S6. Sequencing results hiPSC-CM.

Table S7. Sequencing results heart biopsies.

1.2 Supplementary Figures

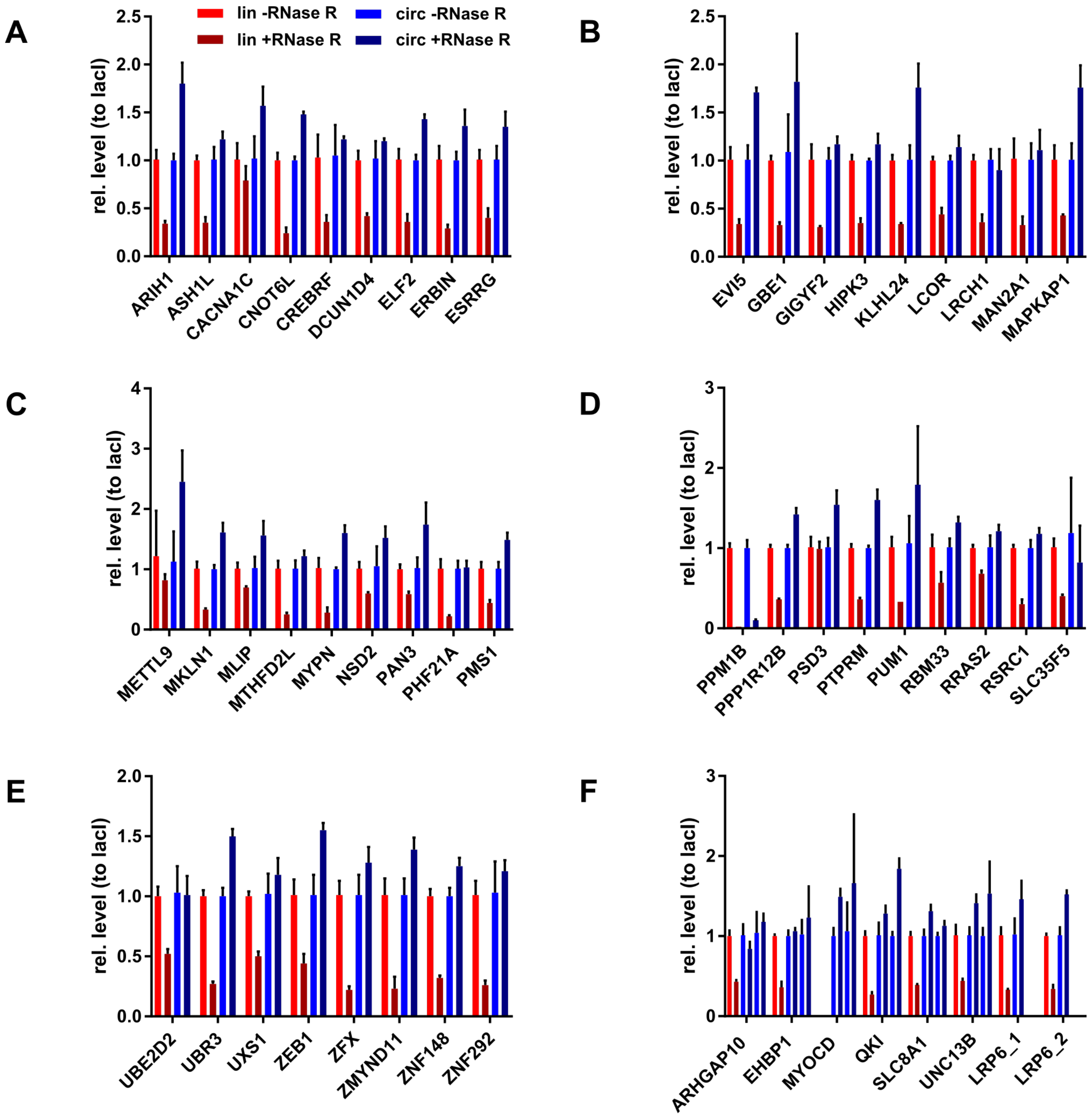

Figure S1. RNase R stability of conserved cardiac circRNAs. **(A-E)** RT-qPCR analysis of circRNA - mRNA pairs in THHR that was either RNase R treated (+ RNase R) or not (-RNase R) as indicated. Red color represents linear RNAs, blue color represents circRNAs. **(F)** Analysis of genes with more than one circRNA as in A-E. For ARHGAP10, EHBP1, MYOCD, QKI, SLC8A1 and UNC13B only the two circRNA isoforms are analyzed by different primer pairs, whereas for LRP6 also different primers for the linear RNAs were chosen.

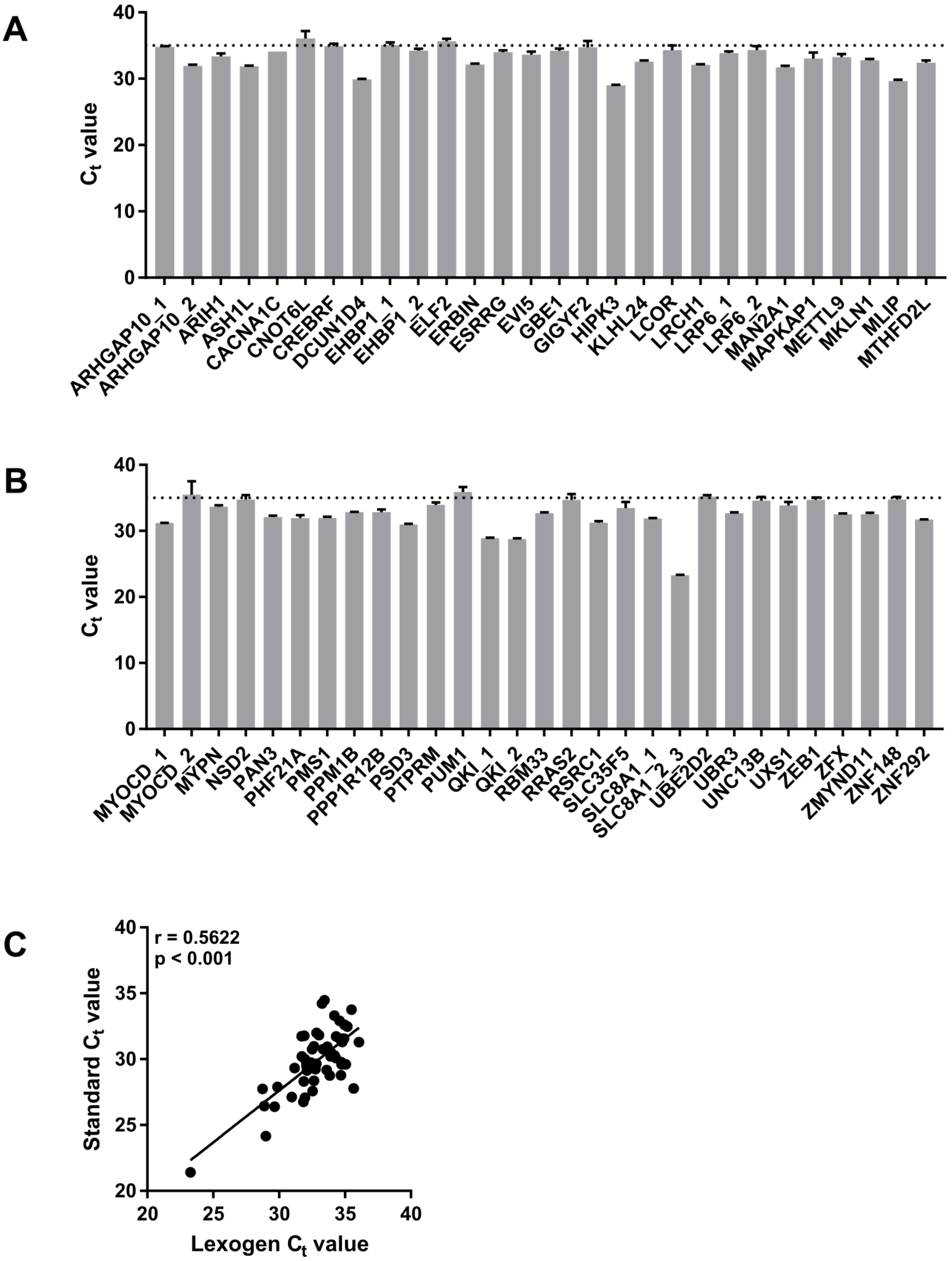

Figure S2. qPCR analysis of Lexo-circSeq libraries. Lexo-circSeq libraries were prepared from 250 ng THHR and analyzed by qPCR with the target-specific RT primer as reverse primer. CircRNAs are presented in alphabetical order. **(A)** Analysis of ARHGAP10 to MTHFD2L circRNAs as indicated. A C_t value of 35 was assumed as detection limit. **(B)** Analysis of MYOCD to ZNF292 circRNAs as indicated. **(C)** Correlation of C_t values derived from qPCR analysis with standard primers (y-axis) and target-specific RT primer as qPCR reverse primer (x-axis). r = Spearman correlation coefficient.

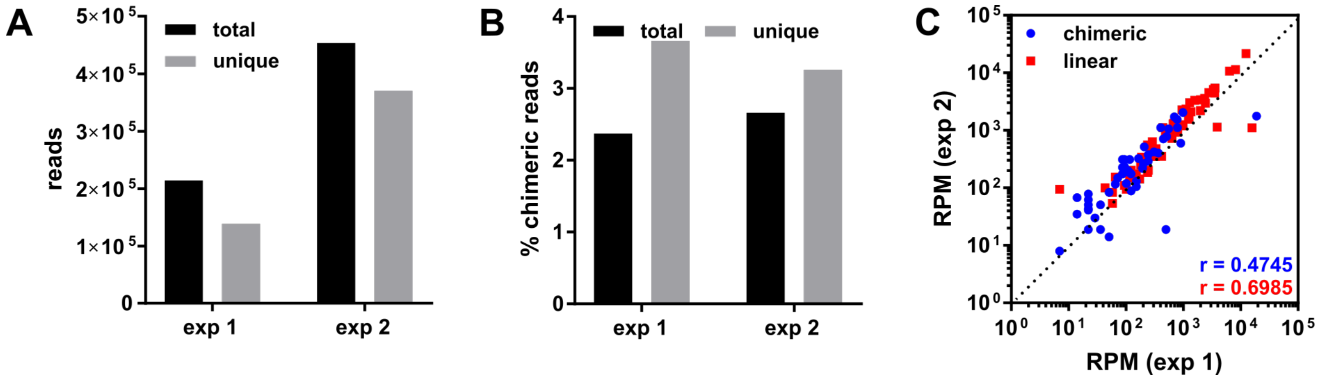

Figure S3. Read statistics of THHR Lexo-circSeq analysis. Two libraries each prepared from 250 ng THHR were sequenced on a Illumina MiSeq instrument and mapped with STAR. **(A)** Number of total reads and uniquely mapped reads. **(B)** Fraction of chimeric reads, calculated for total and uniquely mapped reads in percent. **(C)** Correlation of RPM (uniquely mapped reads) for chimeric and linear reads from experiment 1 and 2. r = Pearson correlation coefficient.

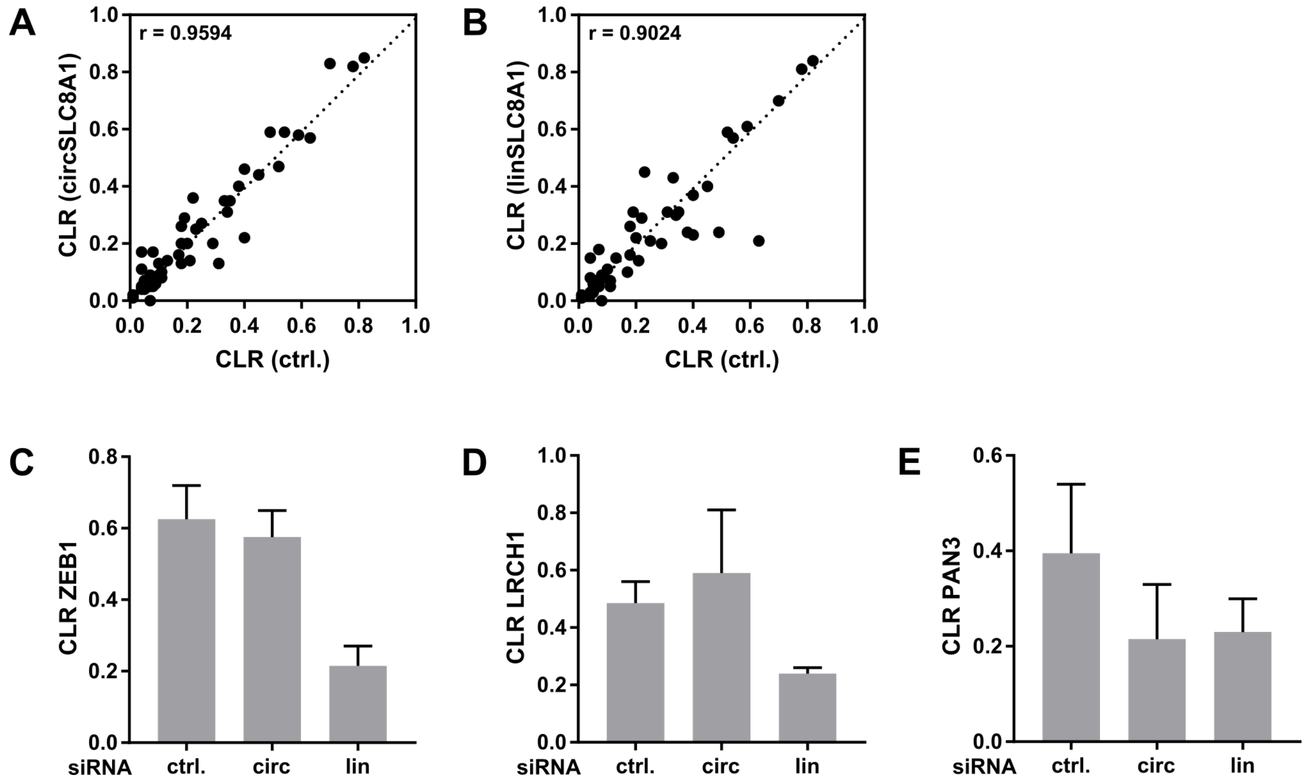

Figure S4. Lexo-circSeq analysis of SLC8A1-depleted hiPSC-CM. **(A)** CLR correlation of ctrl. and circSLC8A1 depleted cells. r = Pearson correlation coefficient. **(B)** CLR correlation of ctrl. and linSLC8A1 depleted cells. r = Pearson correlation coefficient. **(C)** CLR for ZEB1 from Lexo-circSeq analysis as indicated. n = 2. **(D)** CLR for LRCH1 from Lexo-circSeq analysis as indicated. n = 2. **(E)** CLR for PAN3 from Lexo-circSeq analysis as indicated. n = 2.

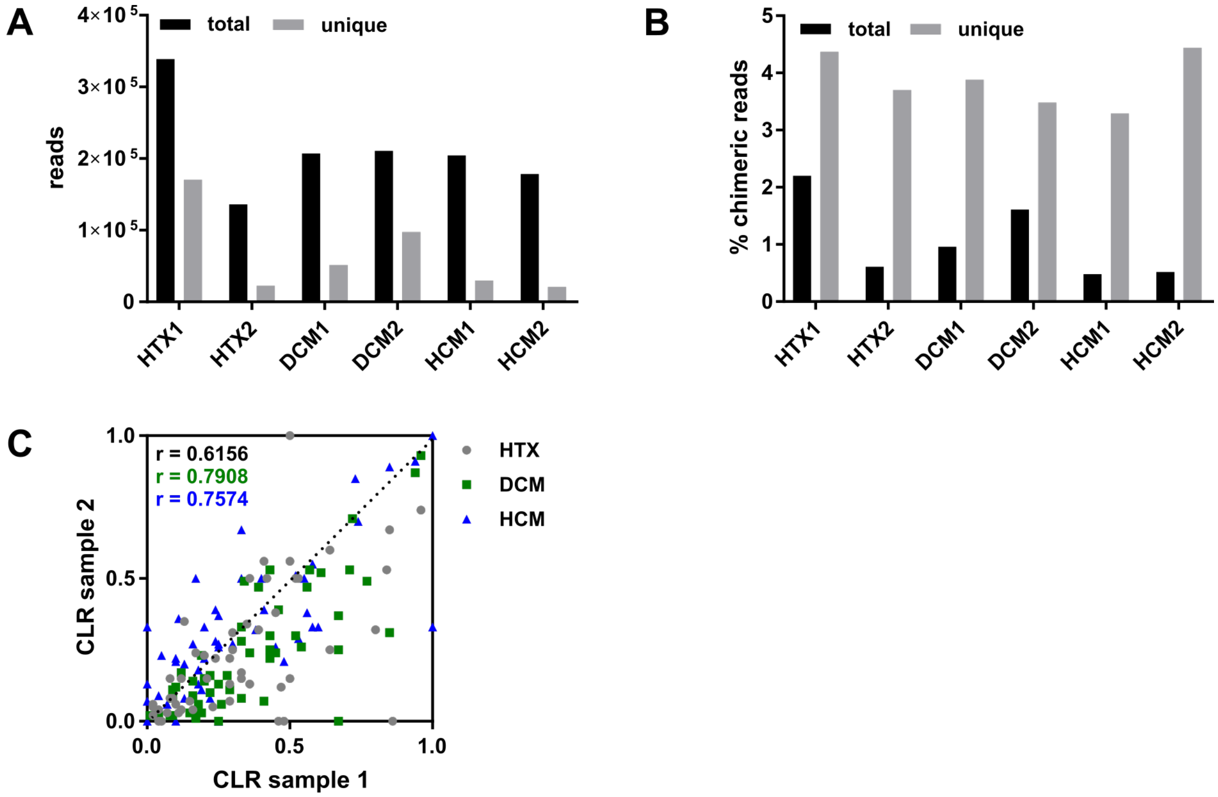

Figure S5. Read statistics of Lexo-circSeq analysis of human heart biopsies. Two libraries each were prepared from biopsies of patients diagnosed with DCM, HCM or heart transplanted patients as control (ctrl.).**(A)** Number of total reads and uniquely mapped reads. **(B)** Fraction of chimeric reads, calculated for total and uniquely mapped reads in percent. **(C)** Correlation of the CLR in pairwise comparisons for the two samples per disease condition. r = Pearson correlation coefficient.
